# Fabrication and Water Treatment Application of Carbon Nanotubes (CNTs)-Based Composite Membranes: A Review

**DOI:** 10.3390/membranes7010016

**Published:** 2017-03-18

**Authors:** Lining Ma, Xinfa Dong, Mingliang Chen, Li Zhu, Chaoxian Wang, Fenglin Yang, Yingchao Dong

**Affiliations:** 1 School of Chemistry and Chemical Engineering, South China University of Technology, Guangzhou 510641, China; 201420120617@mail.scut.edu.cn (L.M.); cexfdong@scut.edu.cn (X.D.); wangchaoxian0620@163.com (C.W.); 2 Key Laboratory of Industrial Ecology and Environmental Engineering (Ministry of Education, MOE), School of Environmental Science and Technology, Dalian University of Technology, Dalian 116024, China; yangfl@dlut.edu.cn; 3 CAS Key Laboratory of Urban Pollutant Conversion, Institute of Urban Environment, Chinese Academy of Sciences, Xiamen 361021, China; mlchen@iue.ac.cn (M.C.); lzhu@iue.ac.cn (L.Z.)

**Keywords:** membrane technology, membrane materials, CNTs-based composite membranes, fabrication methods, water treatment

## Abstract

Membrane separation technology is widely explored for various applications, such as water desalination and wastewater treatment, which can alleviate the global issue of fresh water scarcity. Specifically, carbon nanotubes (CNTs)-based composite membranes are increasingly of interest due to the combined merits of CNTs and membrane separation, offering enhanced membrane properties. This article first briefly discusses fabrication and growth mechanisms, characterization and functionalization techniques of CNTs, and then reviews the fabrication methods for CNTs-based composite membranes in detail. The applications of CNTs-based composite membranes in water treatment are comprehensively reviewed, including seawater or brine desalination, oil-water separation, removal of heavy metal ions and emerging pollutants as well as membrane separation coupled with assistant techniques. Furthermore, the future direction and perspective for CNTs-based composite membranes are also briefly outlined.

## 1. Introduction

Clean water is an essential resource for human life and our ecosystem. However, due to the increasing growth of population and rapid development of economy and urbanization, the increasing pressure on water scarcity issue highly requires cost-effective water treatment technologies to produce high-quality clean water [1,2,3]. On the one hand, seawater and brackish water desalination technologies seem to be the most fundamental approaches that possess great promise to effectively increase clean water supply by producing more freshwater. Since the total sources of seawater and brackish water accounts for almost 98% of all water on the earth, capturing a tiny fraction is expected to impart a huge impact on the issue of fresh water scarcity. On the other hand, recovery and recycling technology of wastewater has become a promising trend in the past decades to achieve water sustainability. Wastewater reuse not only decreases the discharge environmental risk, but also relieves the pressure on fresh water resource shortage [4,5].

Among various technologies for water treatment, membrane separation technology is widely accepted as an emerging route not only to desalinate seawater and brackish water, but also to reuse wastewater [6,7]. Furthermore, due to the advantages offered such as high stability and efficiency, ease operation, low operating cost and capital, low energy consumption and also low pollution, it is known as one of the major technologies for sustainable environmental pollution control engineering. Fundamentally, separation performance of a membrane is mainly limited by its structure and property [8,9]. As far as membrane materials are concerned, both organic polymeric and inorganic membranes have received increasing attentions for water treatment at both laboratory and industrial scales. However, these traditional membrane materials have the limits of a trade-off relationship between selectivity and permeability, and also low anti-fouling property [10]. Therefore, to overcome these critical limitations, new types of membranes need to be designed and fabricated to increase permeability (or flux) and selectivity (or rejection) as well as fouling-resistance.

**Figure 1:**
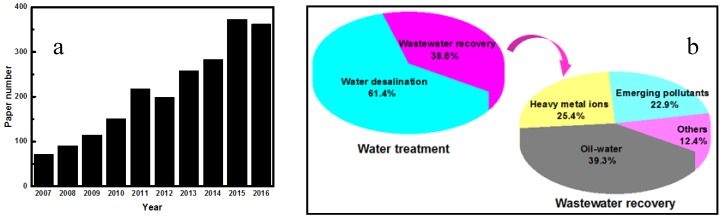
(**a**) Annual number of research papers for CNTs-based composite membranes reported during 2007-2016 (topic keyword “carbon nanotubes composite membranes” searched from Web of Science), all date updated on January 9, 2017; (**b**) Existing water treatment methods for CNTs-based composite membranes.

Recent advances in nanotechnology combined with membrane separation have been recognized as some viable and effective approaches to enhance membrane performance with their synergistic effects for water and wastewater treatment [11]. In particular, carbon nanotubes (CNTs) including single-walled carbon nanotubes (SWCNTs) and multi-walled carbon nanotubes (MWCNTs), owing to their high specific surface area, high mechanical strength, excellent chemical inertness and outstanding water-transport property, have received widespread interests in construction of new composite membranes for water treatment application [12,13,14,15,16,17]. In addition, CNTs exhibit encouraging adsorption, catalytic and electrochemical properties, which are beneficial to couple adsorption, catalytic or electrochemical function with membrane separation process, thus improving water treatment performances of CNTs-based composite membranes. As shown in Figure 1, number of research papers and existing water treatment methods for CNTs-based composite membranes were searched from Web of Science. Obviously, more and more attentions have been paid to the preparation and application of CNTs-based composite membranes from year to year during 2006–2016. In addition, most of CNTs-based composite membranes today are used in water desalination and wastewater treatment. For wastewater treatment, research papers are mainly focused on oil-water separation, removal of heavy metal ions and emerging pollutants. Also, there are some studies on the application of CNTs for treatment of wastewater discharged from membrane manufacturing industry [18].

To date, several review research papers have been published focusing on CNTs-based composite membranes, but these reviews mainly discuss transport and seawater desalination properties of CNTs-based composite membranes [10,14,19]. Therefore, this review summarizes not only fabrication and characterization methods for CNTs and CNTs-based composite membranes via incorporation of CNTs into various membrane materials, but also some popular typical applications for both water desalination and wastewater reuse. In fact, hundreds of research papers are published on the topic annually, as shown in Figure 1, this review discusses the state-of-art some important research papers from the aspects of novelty, typicality and relativity. First, fabrication methods of CNTs are discussed and an overview of the understanding of CNTs’ growth mechanism and characterization techniques is outlined briefly. Then, different fabrication methods of CNTs-based composite membranes are extensively summarized and compared, including chemical vapor deposition (CVD), CVD template method, blending method, in situ polymerization, layer-by-layer self-assembly and direct coating method. In the following sections, the state-of-art applications for water treatment are reviewed, such as water desalination, oil-water separation, removal of heavy metal ions and emerging pollutants.

## 2. Fabrication, Growth and Characterization of CNTs

### 2.1. Fabrication Methods

**Figure 2:**
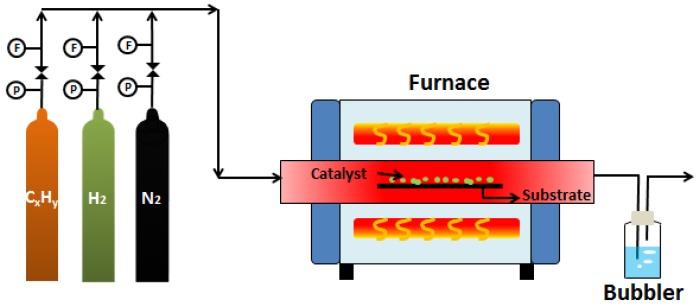
Schematic diagram of the experimental CVD equipment.

Recently, several techniques for CNTs fabrication are available such as electric-arc discharge, laser ablation and chemical vapor deposition (CVD) [20]. Morphology and structure of CNTs can be controllably adjusted by changing process parameters of the currently available fabrication techniques. Among these techniques, CVD is the most popular and widely used due to its relatively simple and flexible process, low set-up cost and easy operation. In addition, CVD appears to be the most potential way to produce high purity CNTs with controllable structure at high yield [20]. CVD is essentially a thermal dehydrogenation reaction whereby a hydrocarbon vapor passes through a tubular reactor in which a transition metal catalyst such as iron, nickel or cobalt is present at high temperature (600–1200 °C) in order to decompose the hydrocarbon [21,22]. A schematic diagram of the experimental set-up used for the growth of CNTs by CVD method is shown in Figure 2 in the simplest form.

### 2.2. Growth Mechanisms

**Figure 3:**
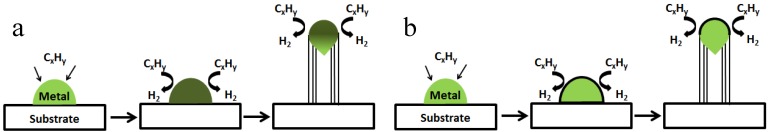
Growth mechanisms for CNTs based on the state of metal catalyst and the diffusion way of carbon: (**a**) VLS mechanism and (**b**) VSS mechanism.

**Figure 4:**
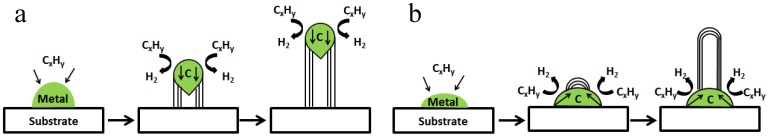
Growth mechanisms for CNTs based on the interaction strength between catalyst particles and substrates: (**a**) tip-growth model and (**b**) base-growth model.

The growth mechanisms of CNTs have been debatable since their discovery. Nevertheless, a widely accepted CNTs growth mechanism can be outlined as follows. Based on the state of metal catalyst and the diffusion way of carbon at high temperature, two different mechanisms including vapor liquid solid (VLS) and vapor solid solid (VSS) are proposed in two general cases, as shown in Figure 3a,b [23,24,25,26]. At high temperature, a carbon precursor is adsorbed and then decomposed on the surface of low-melting-point catalyst nanoparticles to form carbon atoms. The as-generated carbon atoms then dissolve into the bulk of the catalyst nanoparticles to form a liquid metal carbide phase which may decompose into catalyst and carbon atoms at the interface between catalyst and substrate, where carbon atoms are precipitated to form nanotubes. As the reaction proceeds, more carbon atoms are released into the catalyst-substrate interface that allows CNTs to continuously grow [27,28]. This process is known as the VLS mechanism (Figure 3a). VLS mechanism is widely discussed in the literature based on both kinetic data and experimental observation. In the other case, it is well acceptable to propose VSS growth mechanism in CVD process (Figure 3b), which comprises three successive steps. Firstly, carbon precursor decomposes to form carbon atoms. Secondly, carbon atoms spread over the surface of catalyst nanoparticles and move toward the interface between catalyst and substrate. Finally, the carbon atoms precipitate on the interface in the form of CNTs growth by a precipitation-nucleation-crystallization process. On the other hand, according to the interaction strength between catalyst particles and substrates, now there are generally two growth models of CNTs, which involve tip growth model and base growth model [25,26,29], as shown in Figure 4a,b. When the interaction strength between catalyst and substrate is weak, carbon precursor decomposes into carbon atoms on the top surface of a catalyst particle, then carbon atoms diffuse down through the catalyst, and CNTs grow from catalyst bottom, thus pushing the whole catalyst away from the substrate. As long as the catalyst surface is still available for more carbon precursor decomposition, CNTs grow continually to become longer. When the catalyst is fully covered by excess carbon atoms, the CNTs stop growing due to the deactivation of catalyst. This is the tip growth model (Figure 4a). In another situation, when the interaction strength between catalyst and substrate is strong, the decomposition of carbon precursor and the diffusion of carbon atoms are similar to those of the tip growth model (Figure 4b). However, the carbon atoms firstly form a hemispherical dome on the top of the catalyst, which then grow in the form of seamless graphitic cylinder to form CNTs. Hence, the catalyst always fixes on the base to support the growth of CNTs. This is the base growth model.

### 2.3. CNTs Characterizations

**Figure 5:**
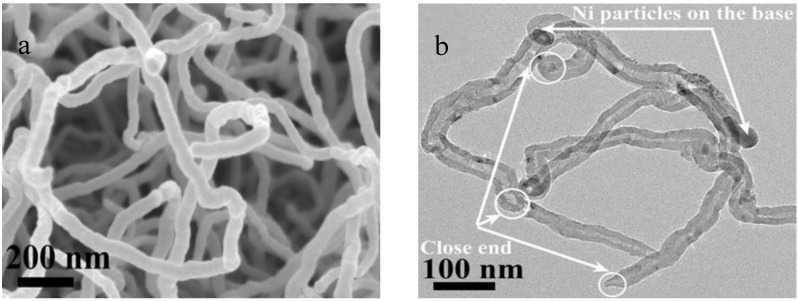
(**a**) SEM image and (**b**) TEM image of CNTs [32].

In order to precisely recognize and analyze the state, structure and property of CNTs, a lot of efforts using various advanced techniques have been made to characterize and analyze CNTs to assess its characteristics and morphologies, as shown in Table 1. Briefly, scanning electron microscopy (SEM) and transmission electron microscopy (TEM) are the well-known techniques that are commonly used to observe the position of tip and sidewall, as well as the morphology of CNTs (as shown in Figure 5) [30,31,32]. Raman spectroscopy (RS) is one of the most powerful characterization techniques for CNTs [33,34,35]. It is routinely employed to evaluate the quality and purity of as-prepared CNTs. A Raman spectrum of CNTs shows two main first order bands including D band and G band. Here the D band is associated with the defects existed in the CNTs, which is observed at around 1300–1350 cm^−1^. The G band is related to the degree of graphitization of CNTs, which is approximately located at 1500–1600 cm^−1^. Hence, the area ratio of the D band and G band (I_D_/I_G_) is usually measured to estimate the level of defects in a specific CNTs sample. For example, the lower I_D_/I_G_ ratio, the lower defects existed in the CNTs. Infrared spectroscopy, X-ray photoelectron spectroscopy (XPS) and thermogravimetric analysis (TG) are usually used to precisely verify the occurrence of functionalization reactions of CNTs for final quality evaluation [36,37]. Therefore, by changing reactants and CVD preparation parameters such as carbon precursor, catalyst, substrate, temperature, pressure, time and gas flow rate assisted with various functionalization and characterization methods, here optimized CNTs could be obtained for various practical applications.

## 3. Fabrication Methods of CNTs-Based Composite Membranes

### 3.1. Functionalization of CNTs

Generally, functionalization of CNTs is a key pre-step for fabrication of CNTs-based composite membranes. On the one hand, due to the inevitable introduction of catalyst, amorphous carbon and other impurities during CVD process, CNTs need further purification and functionalization to remove these impurities. On the other hand, due to the presence of van der Waals force, CNTs tend to form tight bundles which in turn result in the difficulty of CNTs dispersion in most of polymer solvents. Furthermore, the relatively smooth CNTs surface with weak interfacial bonding have limited the loading transmission from polymer matrix or substrates to CNTs, thus reducing the performances of composite membranes. To obtain homogeneously dispersive CNTs in the polymer matrix or substrates and simultaneously activate the CNTs surface, the CNTs are often tailored by creating active functional groups [19,40].

**Figure 6:**
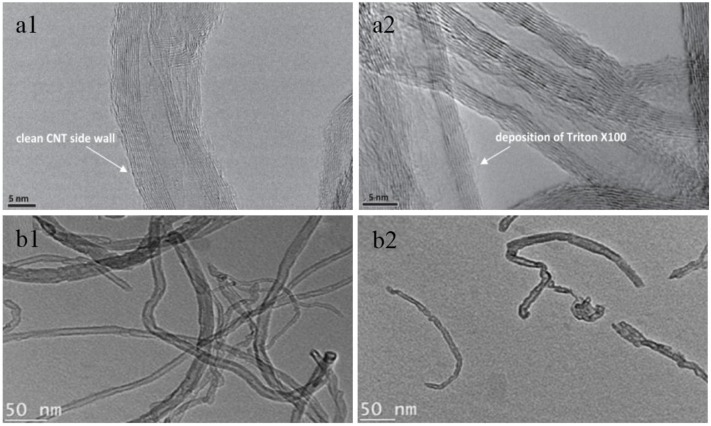
TEM and SEM images of raw CNTs (1) and functionalized CNTs (2) after different functionalization methods: (**a**) non-covalent modification [45] and (**b**) covalent modification [46].

Fundamentally, covalent or non-covalent modifications are used to functionalize CNTs depending on different reaction mechanisms [41,42,43,44]. Covalent modifications are the use of oxidant such as acid or gas to deal with CNTs, thus deliberately grafting functional groups such as carboxyl, hydroxyl and amino groups at the open ends, sidewalls and defect sites of CNTs by covalent bond. In contrast, non-covalent modifications involve adsorption, embedding and winding of surfactants, polymers or large organic molecules onto the surface of CNTs without any effects on the intrinsic structure and property of CNTs, as shown in Figure 6a, which are through non-covalent bonds connection including π-π, hydrogen bond and electrostatic attraction. Indeed, in some cases, covalent modifications with harsh reaction conditions may result in the adversely damages to the structure of CNTs, as shown in Figure 6b, hence the covalent modifications should be carefully carried out in suitable means in order to preserve the properties of CNTs. Both covalent and non-covalent modifications have been used to prepare CNTs-based composite membranes for practical applications. For example, among these various modification methods, using oxidizing acids (e.g., HNO_3_) and strong gas oxidant (e.g., O_3_) tend to be the most prevalent ways in the fabrication of inorganic CNTs-based composite membranes due to the easy removal of amorphous carbon and other impurities from the as-prepared CNTs by a simple operation process. On the other hand, the surface of CNTs used in polymer composite membranes could be modified by both covalent and non-covalent methods, not only to obtain a homogeneous dispersion of CNTs, but also to enhance the interaction between CNTs and polymer matrix or substrates. The functionalization methods of CNTs-based composite membranes are summarized in Table 2.

### 3.2. Fabrication Methods

Considering different types of substrates, CNTs-based composite membranes are mainly divided into inorganic and organic polymeric composite membranes. The fabrication methods of CNTs-based composite membranes include CVD, template method, blending method, in situ polymerization, layer-by-layer self-assembly, direct coating method and so on. Generally, CVD and template method are commonly used to fabricate inorganic CNTs-based composite membranes. Blending, in situ polymerization and layer-by-layer self-assembly are the popular fabrication methods for polymeric CNTs-based composite membranes. The direct coating method is not only employed to fabricate inorganic composite membranes, but also applied to fabricate polymeric composite membranes. Various fabrication methods for CNTs-based composite membranes are reviewed and summarized in Table 3.

#### 3.2.1. CVD Method

CVD is not only an important method for the preparation of CNTs, but also a versatile method for the preparation of CNTs-based composite membranes. The process of CNTs directly grown on the membrane substrates to obtain composite membranes is a similar process to the fabrication of CNTs by CVD. Owing to the high temperature condition of CVD, it is essential for membrane substrates to be quite stable at high temperature. Among various substrates, porous ceramic membranes in different geometries have been widely used to allow CNTs growth due to their excellent chemical, mechanical and thermal stability. Additionally, alumina (Al_2_O_3_), yttria stabilized zirconia (YSZ), and mullite are the most commonly studied membrane materials [47,51,52,53,54,55,56,57]. Interestingly, randomly arranged or vertically aligned CNTs could be fabricated by different CVD conditions. For example, when catalyst precursor and porous Al_2_O_3_ membranes were heated by a two-zone furnace in CVD, vertically aligned CNTs arrays could be deposited on the ceramic substrate. However, when catalyst precursor was preloaded on the Al_2_O_3_ membrane, it is impossible to grow the vertically aligned CNTs arrays [52]. Moreover, Lee et al. developed novel vertically aligned CNTs membranes for water treatment, which fully employed the unique characteristics of CNTs to enhance water permeability. Compared with the highest water permeability of 2400 L·m^−2^·h^−1^·bar^−1^ reported for CNTs membranes, the as-prepared membranes provided a water permeability as high as 30,000 L·m^−2^·h^−1^·bar^−1^ [58]. On the other hand, CNTs were grown in pore channels of a porous Al_2_O_3_ ceramic membrane by CVD leading to a desirable air filtration performance, which breaks the trade-off between retention rate and pressure drop. Compared to the pristine Al_2_O_3_ ceramic membrane, the as-prepared CNTs-Al_2_O_3_ composite membranes showed higher filtration efficiency, while the pressure drop decreased about 62.9% indicating lower energy consumption [54].

CVD has some merits such as relatively simple procedure and easy scaling up, but it is often hard to synthesize size-controlled CNTs with a uniform distribution. Therefore, more attentions have been paid to primarily fabricate a certain template for supporting growth of uniformly-sized CNTs by CVD, named as CVD-template method.

CVD-template method is usually employed to prepare vertically aligned CNTs-based composite membranes with high density, high purity, uniform nanotube diameter and distribution of CNTs. Among various templates, anodic aluminium oxide (AAO) is one of the most commonly used templates for CNTs growth [59,60,61,62,63,64]. Briefly, AAO templates were initially fabricated by electrochemical anodization process. The resulting AAO templates were then immersed into a catalyst precursor solution and placed in flowing hydrogen gas at elevated temperatures in order to form metallic catalyst nanoparticles. Finally, CNTs were vertically grown on the template by CVD at a certain temperature using a carbon source. Therefore, the structure of CNTs mainly depends on that of templates.

**Figure 7:**
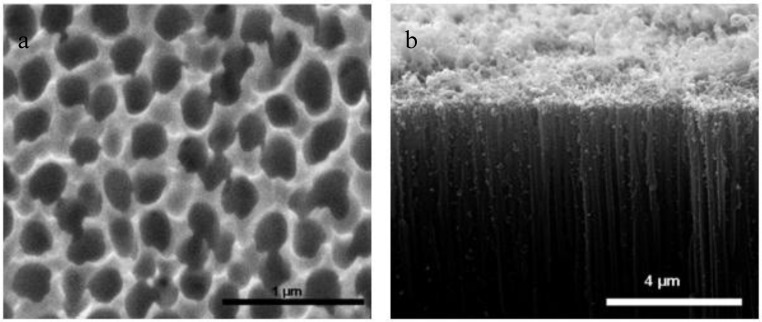
(**a**) SEM image of the AAO template and (**b**) cross-sectional SEM image of the vertically aligned CNTs-AAO [59].

The fabrication of CNTs-AAO with controllable diameter and length of CNTs is demonstrated by Alsawat et al. [48], which indicated that the dimensional features of CNTs-AAO can be precisely engineered by controlling the anodization time of AAO during electrochemical anodization process and the deposition time of carbon source during CVD process. Similarly, vertically aligned CNTs were implanted in the AAO template through CVD with acetylene and nickel severing as carbon source and catalyst, respectively (as shown in Figure 7). The developed composite membranes were examined by adsorption and relative permeability, giving an insight on the factors affecting CNTs growth [59].

#### 3.2.2. Blending Method

In recent years, several researchers successfully have fabricated polymeric composite membranes with CNTs as nano fillers by blending method. Up to now, many polymers have been involved to prepare these CNTs-based composite membranes including polyvinylidene fluoride (PVDF), polyethersulfone (PES), polysulfone (PSF), polyvinyl alcohol (PVA), and polyacrylonitrile (PAN) [65,66,67,68,69,70,71,72,73]. Generally, a homogeneous blending solution was prepared by mixing CNTs and polymers in a suitable solvent through stirring or sonication. Then, polymeric CNTs-based composite membranes were prepared by phase inversion or wet spinning, resulting in flat sheet or hollow fiber geometry, respectively. Obviously, this method is simple with a controllable concentration of CNTs in polymers. For this method, surface modification is a key step with aims of a homogeneous dispersion of CNTs in polymer solvent.

**Figure 8:**
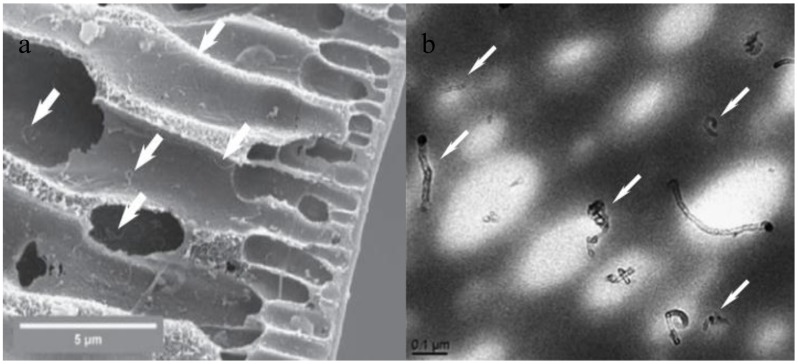
(**a**) SEM image and (**b**) TEM image of CNTs-PES membranes [70].

Majeed et al. modified PAN membranes with hydroxyl functionalized CNTs by a phase inversion process and they found that the mechanical stability and transport properties of the modified membranes were evidently improved by introducing CNTs [72]. Yin et al. demonstrated that the incorporation of oxidized CNTs into PSF membranes could synergistically improve the permeability and anti-fouling ability (as shown in Figure 8) [70]. Similarly, Yang et al. fabricated functional polymer brush grafted CNTs-PES membranes by a phase inversion process. The resulting composite membranes exhibited improved anti-fouling ability, antibacterial activity, and toxin removal ability, which might satisfy diverse separation and purification needs [73].

#### 3.2.3. In Situ Polymerization Method

In situ or interfacial polymerization method can also be used to fabricate polymeric CNTs-based composite membranes with a random arrangement state for CNTs which is similar to blending method. It is now possible to propose a two-step fabrication process, which involves first the blending of CNTs and polymer monomer, then the polymerization of monomer under certain conditions to obtain CNTs-based composite membranes [74,75]. This approach tends to introduce chemical bonds to ensure adequate interfacial adhesion between CNTs and polymers. For example, Kim et al. demonstrated a strategy to prepare polyamide (PA) RO membranes with CNTs [76]. The composite membranes were prepared by interfacial polymerization using trimesoyl chloride (TMC) solutions in *n*-hexane and aqueous solutions of *m*-phenylenediamine (MPD) containing functionalized CNTs. Additionally, the functionalized CNTs were optimized by varying amounts of acid, reaction temperature, and reaction time. The finally optimized composite membranes containing well-dispersed CNTs exhibited high performance (high water flux and salt rejection) and stability. Indeed, although fabrication of polymeric membranes having dispersed CNTs as fillers in the polymer matrixes have been reported quite many times, the polymeric membranes with aligned CNTs for practical water treatment process have not been extensively studied. Recently, Kim et al. developed a novel in situ bulk polymerization method to prepare vertically aligned polymeric CNTs-based composite membranes [77]. Briefly, a vertically aligned CNTs array was infiltrated with polymer monomer followed by in situ polymerization. The polymeric CNTs-based composite membranes showed high gas and water flux, which are comparable to the other vertically aligned CNTs-based composite membranes. Therefore polymeric membranes with aligned CNTs obtained by in situ polymerization method can be potentially used in water treatment applications that may require the specific membranes with high permeability, flexibility and durability.

#### 3.2.4. Layer-by-Layer Self-Assembly Method

Layer-by-layer (LBL) self-assembly method, which can produce charged thin films on molecular levels by adsorbing positive and negative polyelectrolytes incorporating various nanofillers on a substrate via different interactions, including electrostatic, hydrophobic interaction, and hydrogen bonding, provides a tunable and facile method to fabricate membranes with desired properties [78,79]. LBL assembly can precisely control the thickness and compositions of membranes in a comparatively simple and environmental friendly way. To date, only a few studies have been done in the preparation and application of CNTs blended polyelectrolyte membranes for water treatment. Zhang et al. used functionalized CNTs with negatively charged and polyelectrolytes including poly (diallyl-dimethylammonium chloride) and poly (sodium 4-styrenesulfonate) to prepare polymeric CNTs-based composite membranes [80]. By repeating the adsorption steps, homogeneous multilayer CNTs polymer membranes were coated on a PVDF substrate. However, the LBL assembly method has disadvantages such as extended preparation time, repeated operation and use of large quantities of polymers. To overcome these drawbacks, the spray-assisted LBL assembly method has been studied by Liu et al. [81]. In this work, the green solvent, largely composed of deionized water, was used for the fabrication of membranes under spray assistance. Therefore, this work presented a facile way to modify the commercial membrane surface with tuned water flux and enhanced anti-fouling properties.

#### 3.2.5. Direct Coating Method

**Figure 9:**
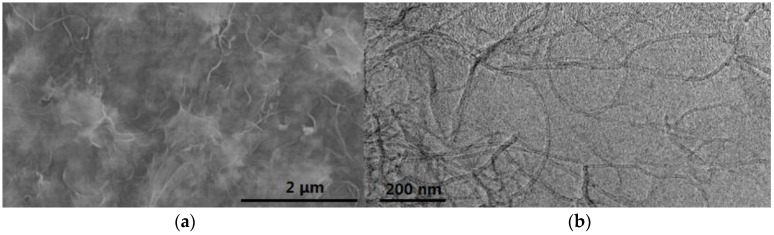
(**a**) SEM image and (**b**) TEM image of CNTs-based composite membrane [87].

Direct coating method not only can fabricate inorganic CNTs-based composite membranes, but also can fabricate polymeric CNTs-based composite membranes. Firstly, CNTs were suspended in water and dispersed by sonication with assistance of surfactants. The resulting suspension was then immediately loaded on cleaned membrane substrates such as PVDF, PES and PA [82,83,84], by filtering, vacuum filtering, syringe-filtering and coating, thus forming a CNTs layer on the substrate surface. Generally, the direct coating method is relatively simple, and capable of forming a CNTs layer on the surface of membrane substrates. For example, Ajmani et al. modified a low pressure membrane (PVDF) with CNTs layer by direct syringe-filtering coating method [82], the resulting membranes showed high resistance to membrane fouling while only slightly reducing the permeability of pure water. Unfortunately, these CNT layers exhibit poor stability due to the weak interaction between CNTs layers and membrane substrates, resulting in the release of CNTs during applications. This release not only greatly restricts their practical value, but also raises important health and safety concerns associated with the presence of CNTs in the treated water. Therefore, Gallagher et al. described a new and simple method to generate backwashable CNTs layer on the inner surface of hollow fiber polymeric membranes [85]. This method included a filtration process of a CNTs suspension through a hollow fiber membrane operating in dead-end filtration system based on the inside-out model. Moreover, Fan et al. reported that graphene-like carbon from polymer pyrolysis can significantly improve the adhesion force between CNTs layer and the substrates. In this work, a vacuum filtration-pyrolysis process was used to construct a CNTs layer on a porous Al_2_O_3_ ceramic membrane. The resulting membranes exhibited excellent stability under rigorous nanoindentation and scratch tests [86]. Recently, Chen et al. reported a novel CNTs-based composite membrane that was fabricated by loading reduced graphene oxide and CNTs onto an AAO membrane via vacuum filtering (as shown in Figure 9). The as-prepared composite membranes showed high permeability and separation performance for drinking water purification [87].

## 4. Water Treatment Applications

CNTs-based composite membranes have been being one popular type of separation membranes for water treatment, since it combines the excellent performances of traditional membrane materials with those of CNTs. To date, some emerging water treatment applications such as water desalination, oil-water separation, removal of heavy metal ions and emerging pollutants in water have been increasingly studied using CNTs-based composite membranes. Therefore, these water treatment applications are extensively discussed in the following sections. A selective overview of fabrication, application and performances of the CNTs-based composite membranes are summarized in Table 4.

### 4.1. Water Desalination

Since the sources of seawater and brackish water account for almost 98% of all water on the earth, capturing a tiny fraction is expected to impart a huge impact on the water scarcity issues. Hence, seawater and brackish water desalination through various desalting technologies seems to be the most straightforward approaches that hold great promise to effectively increase water supply by generating more freshwater from sea and brackish water for the world usage. Membrane separation technology has been widely accepted as a promising route to offer sustainable fresh water in a more economical and green process. At present, CNTs are interestingly investigated and have attracted significant growing attentions due to their properties to enhance the efficiency and capability of currently available membrane processes such as reverse osmosis (RO), nanofiltration (NF) and membrane distillation (MD) [88,89,90,91,92,93].

**Figure 10:**
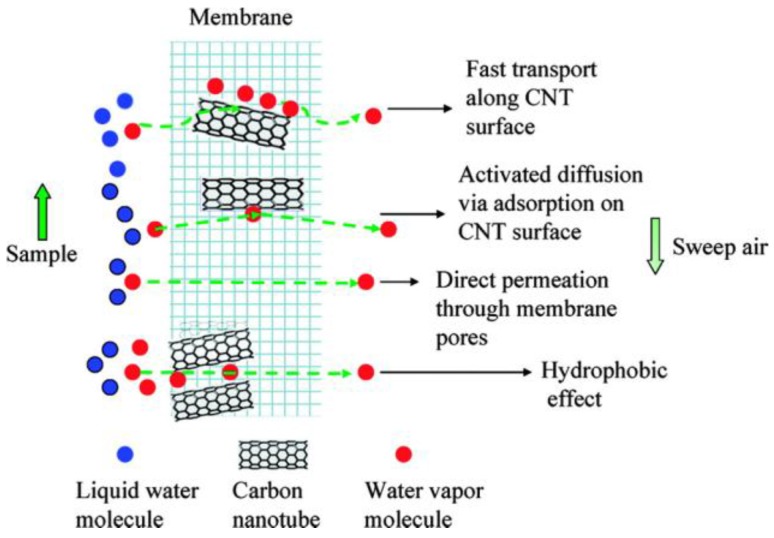
Mechanism of MD of CNTs-PVDF membranes [65].

Son et al. created a novel architecture to produce a better performance thin-film composite (TFC) membrane for seawater reverse osmosis (SWRO) and brackish water reverse osmosis (BWRO) by immobilizing functionalized CNTs in the porous support layer [88]. Beneficial for application, the new membranes showed enhanced water permeability due to their increased hydrophilicity and enhanced pore properties of the support layer without sacrificing NaCl rejection as compared to that of the bare TFC membranes. In SWRO at pressure over 50 bar, the water flux was enhanced 10%–20%, and a 90% enhanced water flux was achieved in the BWRO operating pressure at 30–40 bar. Recently, CNTs-PES mixed matrix membranes have been prepared for NF application. With incorporation of CNTs, the as-prepared membranes showed higher flux and salt rejection than the PES membranes [89]. This study found that when CNTs concentration reached 0.1 wt %, the CNTs-PES membranes obtained the highest water flux (38.91 L·m^−2^·h^−1^) and Na_2_SO_4_ rejection (87.25%) at 4 bar. In addition, Gethard et al. demonstrated that CNTs can serve as a sorbent to provide an additional pathway for solute transport in the CNTs-PVDF membranes, as shown in Figure 10. Compared to traditionally used MD membranes, the CNTs-PVDF membranes can improve MD process at a relatively lower temperature and higher permeate vapor flux for a wide range of salt concentration up to the equivalent of seawater [65]. Therefore, these may lead to a more economical MD process which could compete with the existing desalination technologies such as RO and NF.

### 4.2. Oil-Water Separation

**Figure 11:**
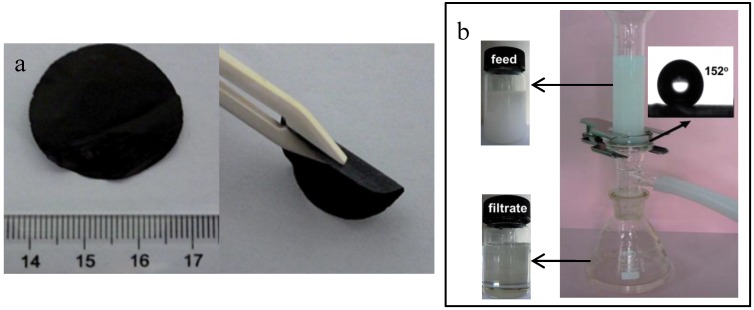
Oil-water separation for superhydrophobic CNTs-polystyrene composite membranes: photographs of (**a**) membranes and (**b**) oil-water separation [95].

High volumes of wastewater in the form of oil-water emulsion are produced in various sources such as crude oil production, oil refining, transportation, domestic sewage and others. Discharging oil-water emulsion wastewater into water can cause severe damage to the environment and serious harm to human health. Membrane filtration including microfiltration (MF), ultrafiltration (UF), and nanofiltration (NF) has been widely applied for oil-water treatment with distinct advantages of high quality water produced, low energy consumption and small footprint [101,102,103]. However, the difficulty is resulted from the deformable nature of oil droplets, which renders the only use of size exclusion separation ineffective for fully removing oil from water. Fundamentally, due to the hydrophobicity and adequate adsorption sites for oil molecules in water of CNTs, assimilation of adsorption into size exclusion separation membrane has been proposed as an effective way to treat oil-in-water emulsion. Chen et al. fabricated a new CNTs-based composite membrane by implanting CNTs in pore channels of an Y_2_O_3_-ZrO_2_ (YSZ) membrane via CVD [56]. The YSZ membranes with CNTs showed 100% rejection rate of oil particles and maintained a permeate flux of 0.6 L·m^−2^·min^−1^ at 1 bar pressure drop over 3 days of operation. This improved performance was attributed to the formation of lipophilic soft layers on CNTs, which could exhibit both adsorption and size exclusion properties as compared with only size exclusion separation for YSZ membranes. Generally, polymeric membranes are unpractical for removing oil from water because polymeric membrane materials are in some cases vulnerable to oil molecules, which will cause the deformation and degradation of membrane structure membrane property. A novel CNTs polymer composite membrane was prepared to separate oil from water for treatment of oil-containing wastewater [94]. Due to the integration of CNTs, the mechanical properties such as tensile strength, Young’s modulus, and toughness were increased indicating the suitability and good durability of the composite membranes in oil-water treatment. Moreover, Gu et al. [95] demonstrated a facile and versatile approach to prepare superhydrophobic CNTs-polystyrene composite membranes for emulsified oil-water separation, as shown in Figure 11. The resulting membranes could effectively separate a wide range of surfactant stabilized water-in-oil emulsions with a high rejection efficiency (>99.94%) and a promising flux (5000 L·m^−2^·h^−1^·bar^−1^).

### 4.3. Heavy Metal Ions Removal from Wastewater

Wastewater from many industries such as metal plating facilities, mining operation, chemical manufacturing, and battery industries contains one or more toxic metal ions [104]. Recently, CNTs have been used as promising adsorbents for adsorption of metal ions such as Pb^2+^, Zn^2+^, Ni^2+^, Sr^2+^, Cu^2+^, Cd^2+^, Co^2+^ and Cr(VI) [105,106]. In particular, the current challenge is how to convert the excellent adsorption capability of CNTs into a cost effective and valuable filtration membrane for specific applications. Based on this, Parham et al. demonstrated a highly efficient and versatile CNTs-based composite filter for the removal of heavy metal ions from water [96]. The composite membranes appeared to approximate 100% heavy metal ion removal efficiency. Similarly, Tofighy et al. also fabricated a novel adsorptive membrane for Ni^2+^ ions removal from water by implanting CNTs in pore channels of ceramic membrane substrates via CVD [47]. The adsorption behavior of the membrane matched well both Langmuir and Freundlich isotherm models. However, due to the relatively large pore size of the new ceramic CNTs-based composite membranes, the membranes only took advantage of adsorption ability of CNTs without using membrane filtration effect for metal ion removal. Therefore, the efficiency of membranes was low in a continuous filtration operation model, which is not viable for practical application. Shah et al. synthesized functionalized CNTs-PSF composite membranes by the phase inversion method [97]. The presence of CNTs effectively imparted the membranes excellent adsorptive nature, and reduced pore size to the range of 20–30 nm, thus helping the membranes to be efficient enough for metal ion removal under continuous filtration operation. For example, the composite membranes exhibited a 94.2% removal for Cr(VI) and 78.2% removal for Cd^2+^, just 10.2% and 9.9% for the bare PSF membranes, respectively.

### 4.4. Removal of Emerging Pollutants from Wastewater

Emerging pollutants, such as pharmaceutical and personal care products (PPCPs), persistent organic pollutants (POPs), and environmental endocrine disruptors (EEDs), exist in the environment with low concentration (ng·L^−1^ ~ µg·L^−1^), but tend to cause serious harm to ecology and human health [107,108]. Therefore, the removal of emerging pollutants in water or wastewater has received more and more attentions in recent years. Interestingly, a novel CNTs-PVDF composite membrane was fabricated and used in the filtration of triclosan (TCS), acetaminophen (AAP) and ibuprofen (IBU) to determine the capabilities and mechanisms of PPCPs removal [98]. The removal rates ranged from approximately 10% to 95%, and increased with PPCPs with more aromatic rings (AAP ≈ IBU < TCS), CNTs with lesser surface oxygen content (oxidized MWCNTs < MWCNTs) and with more specific surface area (MWCNTs < SWCNTs). In addition, considering relatively low initial concentration of PPCPs in natural water or municipal wastewater, the CNTs-based composite membranes are expected to be capable of removing PPCPs for prolonged period of separation time, without the needs for regeneration, which is important to practical application.

### 4.5. Membrane Separation Coupled with Assistant Techniques

**Figure 12:**
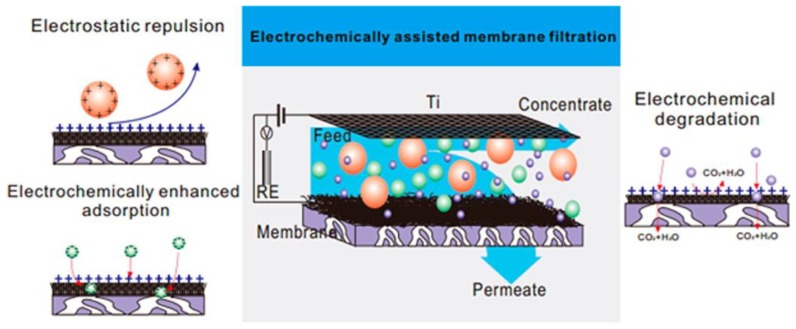
The separation mechanism of CNTs-Al_2_O_3_ composite membranes under electrochemical assistance [86].

Due to the excellent adsorption, electrochemical and catalytic properties of CNTs, the integration of membrane filtration with these unique CNTs properties may be an alternative way for removal of pollutants by using CNTs-based composite membranes. Under electrochemical assistance, CNTs-Al_2_O_3_ composite membranes for the removal of phenol have been intensively studied by Fan et al. [86]. The membrane presented low phenol removal efficiency under the absence of electrochemical assistance process. In contrast, under +1.5V electrochemical assistance, the removal efficiency of the membrane increased to nearly 100%. These results indicated that the separation performance of CNTs-Al_2_O_3_ composite membranes can be significantly improved by electrochemical functions, such as electrostatic repulsion, adsorption and degradation, as illustrated in Figure 12, which provide a new insight for design of CNTs-based composite membranes for pollutants removal applications. Recently, CNTs-based composite membranes have been studied for assimilation of photocatalysis and ozonation catalysis into membrane filtration for water treatment, all of these results can extend the composite membranes for various practical applications [99,100]. For example, Oulton et al. fabricated CNTs-AAO composite membranes for *para*-chlorobenzoic acid (p-CBA) chemical ozonation in a dead-end filtration unit, which integrates adsorption, ozonation catalysis and membrane filtration [100]. The *p*-CBA removal rates ranged from 20% to 50%, and increased with higher ozone concentration and mass of CNTs.

## 5. Summary

Currently, polymeric or inorganic membrane separation processes might have reached a threshold at which it is hard to improve separation performances only via further optimization of membrane itself. The combination of nanotechnology and membrane separation offers new approaches to overcome the challenge.

This review provides an overview of CNTs-based composite membranes from fabrication, characterization and functionalization of CNTs to fabrication methods and water treatment applications of CNTs-based composite membranes. Fundamentally, high purity CNTs for fabrication of composite membranes can be prepared by CVD due to its easy scaling-up production and low cost via the widely accepted CNTs growth mechanism. Generally, characterization and further functionalization of CNTs can be actively carried out in order to analyze and obtain desirable and tunable characteristics. The presence of functional groups at the open ends, sidewalls and defect sites of CNTs is capable of not only improving the dispersion of CNTs for membranes fabrication process, but also increasing composite membranes performances. In addition, among various fabrication methods, CVD is the most popular fabrication method for inorganic (ceramic) CNTs-based composite membranes while the blending method is quite flexible for polymeric CNTs-based composite membranes. Furthermore, compared to bare membranes (polymeric and inorganic membranes), CNTs-based composite membranes have remarkably improved performances in terms of separation and purification capabilities for various water treatments.

Since the research for CNTs-based composite membranes is still at the premature stage, and many problems are yet to be resolved while more challenging issues need to be dissolved. For example, it is difficult to compare the characterization information presented by different research papers on the as-prepared CNTs-based composite membranes using different characterization methods. Moreover, while CNTs-based composite membranes have been successfully fabricated through different methods, there are still limitations for control of fabrication parameters, such as controllable loading of CNTs, and more especially scale-up manufacturing. Therefore, it is necessary to extensively continue research of CNTs-based composite membranes regarding characterizations and fabrication processes. Moreover, the selection and applications of CNTs-based composite membranes should be considered not only from the view point of water treatment performances, but also from that of potential toxicity effects when CNTs are released to the environment in some cases. Thus, such studies for example, how to sufficiently enhance adherence between CNTs and substrate (matrix), possibly deserve more attentions in the near future. Also, some novel approaches to composite membrane formation using CNTs should be developed to enhance membranes mechanical properties such as biomimetic design [109]. In addition, although membrane separation coupled with other assistant techniques (such as adsorption, catalysis and electro-chemistry) is available, a deep understanding of the mixed mechanisms for such CNTs-based composite membranes is still lacking. Therefore, more scientific and technical inputs are needed to investigate from the aspects of water chemistry, membrane process parameters, properties of both CNTs and composite membranes under specific conditions. There is no doubt that CNTs-based composite membranes will find their role in the future direction of desalination and purification technology for more and more water treatment applications to allow for greater flexibility and broader perspective in addressing critical water issues.

## Figures and Tables

**Table 1:** Characterization techniques of CNTs.

Characterization Techniques	Major Aims	References
SEM	Morphological analysis (diameter, length, defects and purity), arrangement state	[30]
TEM	Morphological analysis (diameter and defects) Characterization SWCNTs and MWCNTs MWCNTs internal microscopy (number of layers and distance between them)	[30]
Energy dispersive spectroscopy (EDS)	Elemental composition, functionalization	[31]
RS	Characterization SWCNTs and MWCNTs Quality and purity SWCNTs mean diameter and chirality	[33]
Fourier transform infrared spectroscopy (FT-IR)	Functionalization	[31]
Ultraviolet-visible spectroscopy (UV-Vis)	CNTs diameter, length and purity Dispersion state	[30]
Fluorescence spectroscopy (FS)	Semiconducting SWCNTs diameter and chirality	[38]
XPS	Elemental composition, functionalization	[36]
TG	Purity, functionalization	[37]
Boehm titration	Number of oxygen-containing functional groups	[39]

**Table 2:** Functionalization of CNTs.

Composite Membranes	Functionalizations	Major Aims	References
CNTs-mullite	HNO_3_	Incorporate acidic functional groups Decrease impurities Increase membrane performances	[47]
CNTs-AAO	H_2_O_2_	Incorporate functional groups Increase hydrophilicity	[48]
CNTs-PES	H_2_SO_4_:HNO_3_ (3:1)	Incorporate functional groups Shorten the length of CNTs Increase CNTs dispersion and interfacial bonding	[46]
CNTs-PA	O_3_	Increase sidewall functionalities Shorten the length of CNTs Increase CNTs dispersion Reduce biofouling	[49]
CNTs-PVA	Chitosan	Intact structure of CNTs Increase CNTs dispersion Improve the packing structure	[50]
CNTs-polyetherimide	Surfactant	Incorporate surfactants Intact structure of CNTs Enhance membrane thermal stability and mechanical strength	[45]

**Table 3:** Fabrication methods of CNTs-based composite membranes.

Properties	CVD	CVD- Template	Blending	In situ Polymerization	LBL Assembly	Direct Coating
Membrane materials	Inorganic	Inorganic	Polymeric	Polymeric	Polymeric	Inorganic or polymeric
Interfacial bond	In situ growth	In situ growth	Hydrogen bonding, Van der Waals forces, etc.	Covalent bonds	Covalent bonds, electrostatic, etc.	Covalent bonds
Arrangement state	Aligned or random	Aligned	Random	Aligned or random	Aligned or random	Random
Stability	Excellent stability	Excellent stability	Good stability	Poor mechanical stability	Good stability	Good stability
Defects	Impurities	Impurities	Difficulty in dispersion	Poor stability	Pinholes	Easy detachment
Membrane area	Limitation to substrate	Limitation to template	No limitation	Small	No limitation	Limitation to substrate
Membrane thickness and pore size	Controllable	Controllable	Controllable	Controllable	Controllable	Controllable
Operability	Easy	Complicated	Easy	Complicated	Complicated	Easy
Practicability	Strong	General	Strong	General	General	Strong

**Table 4:** An overview of fabrication, application and performances of the CNTs-based composite membranes.

Membrane Material	Fabrication Method	Application	Membrane Performances		References
			Permeability (L·m^−2^·h^−1^·bar^−1^)	Selectivity	
CNTs-TFC	Phase inversion	RO	1.2	96.1% (2000 ppm NaCl, 20 bar)	[88]
CNTs-PES	Phase inversion	NF	9.7	87.3% (200 ppm Na_2_SO_4_, 4 bar)	[89]
CNTs-PVDF	Direct coating	NF	5.0	50%–60% (200 ppm Na_2_SO_4_, 4 bar)	[90]
GO coated VA CNTs	CVD	NF	5.0	44.9% (10 mM NaCl, 15.5 bar)	[91]
PA coated VA CNTs	CVD	NF	4.0	64.8% (10 mM NaCl, 15.5 bar)	[91]
CNTs-PP	Blending	MD	36.8 (L·m^−2^·h^−1^)	99.9% (10,000 ppm NaCl)	[92]
CNTs-PTFE	Blending	MD	69 (L·m^−2^·h^−1^)	(34,000 ppm NaCl)	[93]
CNTs-PVDF	Blending	MD	19.1 (L·m^−2^·h^−1^)	93% (88 ppm NaCl and 12 ppm MgSO_4_)	[65]
CNTs-YSZ	CVD	MF	36	100% (210 ppm oil emulsion)	[56]
CNTs-PSF	Phase inversion	UF	47.5	97.4% (287 ppm oil emulsion, 4 bar)	[94]
CNTs-polystyrene	Photografting	UF	5000	>99.9% (oil emulsion)	[95]
CNTs-ceramic	CVD	Adsorption		99.9% (12,000 ppm CuCl_2_)	[96]
CNTs-mullite	CVD	UF	8.1	62.9% (100 ppm Ni(NO_3_)_2_, 4 bar)	[47]
CNTs-Al_2_O_3_	CVD	UF	>4.5	>75% (100 ppm Cu(NO_3_)_2_ at pH 6, 4 bar)	[55]
CNTs-PSF	Phase inversion	UF		94.2% (1 ppm K_2_Cr_2_O_7_ at pH 2.6, 4.9 bar)	[97]
CNTs-PVDF	Direct coating	MF-adsorption		10%–95% (1 ppm PPCPs)	[98]
CNTs-Al_2_O_3_	Direct coating	MF-electrochemistry		100% (5 ppm phenol, +1.5 V)	[86]
CNTs-TiO_2_/Al_2_O_3_	Direct coating	MF-photocatalysis	290	90% (10 ppm HA, 1 bar)	[99]
CNTs-AAO	Direct coating	MF-catalytic ozonation		20%–50% (2 µM p-CBA and 160 µM O_3_ at pH 7)	[100]
